# Usability and Adoption of Smartwatches by Older Adults in Bangladesh: User Study

**DOI:** 10.2196/76404

**Published:** 2026-06-30

**Authors:** Fariah Mahzabeen, Fazlea Rabby, Neazmul Mowla, Sadia Afra Ibnat

**Affiliations:** 1 ECE, North South University, Plot 15, Bashundhara, Dhaka, 1229, Bangladesh, 880 1312052160; 2 ECE, Alumni, Research Assistant, North South University, Dhaka, Bangladesh

**Keywords:** smartwatch, wearables, older people, usability, user interface, age-appropriate design, socioeconomic influences, human-computer interaction, low- and middle-income countries

## Abstract

**Background:**

Wearables such as smartwatches can support point-of-care health management for older adults while reducing pressure on health care systems as aging populations grow. Although many studies emphasize technical accuracy, user-centered research on smartwatch adoption among older adults remains limited, particularly in low- and middle-income countries, such as Bangladesh.

**Objective:**

This study evaluated real-world usability, acceptance, and adoption of smartwatches among socioeconomically diverse older adults (aged ≥60 y) in a low- and middle-income country context. It examined how age, education, socioeconomic background, and cultural perceptions influence user experience, and identified barriers and facilitators related to comfort, usability, and long-term engagement. It also explored health-related features that older adults perceived as missing.

**Methods:**

Participants were recruited from older adult communities with diverse socioeconomic and educational backgrounds, and received a commercially available smartwatch. A mixed methods, 3-phase longitudinal design was conducted, including a short-term survey (n=27), 3 long-term survey data points (n=19, 18, and 18), and 2 focus group interviews (n=13 and 12). Quantitative analyses included paired *t* tests across longitudinal data points and chi-square tests for distributional associations, complemented by thematic qualitative analysis.

**Results:**

Comfort improved substantially over time, with participants rating the smartwatch as very comfortable, increasing from 26% (5/19) at baseline to 80% (16/20) at final follow-up. Heart rate monitoring remained the most frequently used feature, rising from 68.42% (13/19) to 75% (15/20). Paired *t* tests showed no significant longitudinal changes in daily usage (*t*_5_=0.00 to 0.22, *P*=.83 to ≥.99), comfort (*t*_4_=−0.07 to 0.30, *P*=.78 to .95), most liked features (*t*_8_=−0.63 to 0.51, *P*=.30 to .62), or clarity of instructions (*t*_2_=0.00 to 0.19, *P*=.87 to ≥.99). Significant increases were observed in feature usage (data point [DP] 2 vs DP3: *t*_8_=−4.16, *P*<.001; DP1 vs DP3: *t*_8_=−2.53, *P*=.03) and perceived difficulty of use (DP2 vs DP3: *t*_8_=−4.29, *P*<.001; DP1 vs DP3: *t*_8_=−2.31, *P*=.05). Chi-square analyses indicated a significant association between study phase and clarity of instructions (*χ*^2^_4_=10.3, *P*=.03), with a trend for comfort (*χ*²_8_=13.8, *P*=.08); other usability dimensions were non-significant (*χ*_²16_=7.1 to 17.5, *P*=.35 to .97). Socioeconomic differences emerged in data-sharing preferences, with higher socioeconomic participants favoring continuous sharing and lower socioeconomic participants preferring emergency-only sharing.

**Conclusions:**

Older adults can adapt to wearable health technologies when given adequate time and support, regardless of education level. Preferences for a limited set of essential health features and faster adoption among participants with lower educational backgrounds highlight the need for simpler smartwatch designs. These findings can guide age-appropriate wearable technologies that prioritize usability, comfort, and familiarity to improve health care access in resource-limited settings.

## Introduction

The use of wearables for point-of-care health management has the potential to enhance independence and quality of life among older adults while alleviating pressure on health care systems amid an aging population worldwide [1-3]. While numerous studies have examined the technical accuracy and performance of wearable technologies, significant challenges and limitations remain [4]. Validation studies have assessed wearable activity trackers in older and clinical populations, supporting measurement reliability while highlighting the need for usability-focused research [5]. Furthermore, user-centered investigations into factors influencing the adoption of wearables among older adults, particularly in low- and middle-income countries (LMICs), such as Bangladesh, are scarce [6].

Wearables have demonstrated benefits in chronic disease management, such as diabetes care, through real-time monitoring and personalized feedback [7]. Wearable sensors and systems have also been extensively explored in rehabilitation and long-term monitoring, demonstrating value for supporting functional health needs [8]. Smartwatches and Internet of Things–based applications can also facilitate independent living by enabling safety monitoring and home health supervision for aging populations [9]. Evidence also suggests that wearable-based interventions can improve physical activity and health outcomes among older adults in community settings [10]. Wearables offer diverse applications, including position and posture tracking, activity recognition, and real-time vital sign monitoring, which are particularly useful in home health care [11].

Despite these advantages, adoption among older adults is inconsistent, and mixed methods studies suggest that usability, contextual, and behavioral factors shape long-term engagement [7]. Design aspects may introduce stigma perceptions, affecting willingness to adopt such devices [12]. Acceptance models further indicate that perceived usefulness and health-monitoring value are central determinants of adoption intention [13].

Older adults may also experience technology anxiety and resistance to change, reducing their readiness to adopt wearable health technologies [14]. Predictive studies suggest that adoption is influenced by multiple interacting antecedents, including behavioral, psychological, and contextual factors [15]. Concurrently, wearable health technologies intersect with artificial intelligence–driven markets, where health consciousness and privacy preferences play important roles in user adoption [16].

Community-based research indicates that older adults’ acceptance of smartwatches is often driven by health and location tracking functions when these align with personal needs [17]. Adoption of fitness technology encourages physical activity and behavioral change, but adoption and long-term engagement are influenced by both perceived usefulness and social visibility, highlighting the interplay between practical value and social context [18,19]. User-centric research from LMIC settings, such as the Philippines, reinforces that real-world adoption depends on usability, perceived value, and local contextual factors [20,21]. At the same time, wearable technology development faces broader challenges, including reliability, battery life, and interface complexity, which can hinder adoption [21].

Older adults often encounter difficulties using smartwatches, including small displays, discomfort, limited battery life, and complex interfaces, making usability evaluation critical [22]. Qualitative studies emphasize that long-term comfort is shaped by interacting human and design factors and strongly influences continued use [23,24]. Focus group research suggests that perceptions, motivators, and barriers influence tracker use during behavior change maintenance, emphasizing the role of perceived value and ongoing support [24]. Technology adoption decisions are further shaped by psychological, social, and situational factors [25].

It is found that qualitative synthesis methods are effective for exploring lived experiences and uncovering usability barriers that quantitative approaches may overlook [26]. Observational studies show that wearable activity trackers can be acceptable for older adults in real-world settings, although practical challenges remain [27]. Systematic reviews highlight that experiences with wearables vary depending on device type, setting, support systems, and integration into daily routines [28,29]. For example, field testing of fall detection wearables demonstrates that real-world deployment can reveal practical challenges not captured in controlled studies [29]. Wearable systems for measuring gait and physical activity in chronic conditions require usability considerations to ensure adherence and data quality [30,31].

Evidence suggests that continued wearable use among older adults is strongly associated with comfort, alignment with daily routines, and perceived value, underscoring the importance of context-sensitive, user-centered design [23,32-38]. Addressing these factors is essential for designing age-appropriate wearable technologies that maximize adoption, usability, and health impact in resource-limited contexts. Hence, in this work, we use a mixed methods study to examine user adoption and reactions to existing smartwatches across diverse older adult populations, and to evaluate age-centric, socioeconomic, and user-focused adoption barriers and facilitators.

## Methods

### Recruitment

#### Overview

**Figure 1.**
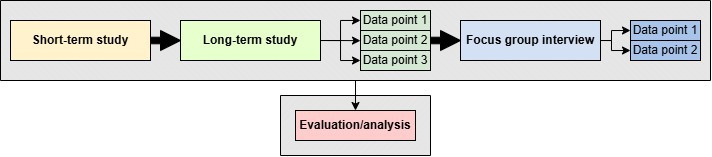
Overall methodology showing the study design phases: short-term, long-term (with 3 data points), and focus group interviews (with 2 data points), followed by evaluation and analysis.

The core goal of this study is to assess the usability of smartwatches among the older adult communities in an LMIC, like Bangladesh. The first step of the study was to identify interest levels and engage with communities of older adult individuals, as detailed in the following sections. Following that, a survey process of 3 cycles was conducted using purposive sampling techniques by recruiting individuals from different socioeconomic and educational backgrounds, lifestyles, and educational backgrounds. This study cycle includes (1) a short-term survey, (2) a long-term survey with 3 data points (DPs), and (3) a focus group interview with 2 DPs. The final step of the study includes survey analysis and finding key insights from all the cycles for designing a specific older adults–age-centric health monitoring smartwatch. Figure 1 presents a flowchart of the methodological framework of the study.

#### Participant Demographics and Recruitment

An institutional review board (IRB) was approved by the North South University Scientific Regulatory Committee, and all participants were asked to complete a consent questionnaire. At the start of our study, we surveyed older adult individuals aged 60 years and older from our local community (friends, families, neighbors, and service staff) to understand their interest in using smartwatches for health monitoring. Then we contacted 5 old-age homes in and around Dhaka City and visited them to assess the interest level of participation. Initially, 2 old-age homes and a group of janitors (from residential areas of the authors) agreed and were recruited in the short-term survey study. However, due to internal management issues at one of the old-age homes, the consecutive surveys were conducted with the participants of one old-age home called Probeen Nibash (managed by the Ministry of Social Welfare in Dhaka, Bangladesh). We procured approval from the management of Probeen Nibash to conduct the study at their center and then talked individually to residents to explain the purpose and format of the long-term study. Based on their consent and the inclusion-exclusion criterion, selective participants were recruited from this old-age home. Similarly, several janitors were approached from the authors’ local communities, and those who willingly consented were recruited to continue the long-term study. The participants were between 60 and 103 years old, male and female, and purposefully sampled from diverse backgrounds. The participants were divided into 2 groups based on socioeconomic and educational backgrounds.

#### Inclusion and Exclusion Criteria

Older adults (older than 60 y) who were generally healthy and able to walk >50% time around on their own or with the use of an assistive device (such as a walker) were included. Older adults with restricted mobility (like in a wheelchair >50% time) or conditions that may limit the use of devices, such as blindness and cognitive decline, were excluded.

### Survey Method and Participant Demographic Analysis

#### Overview

Before the first survey, a preliminary questionnaire session was conducted with each participant to collect demographic and baseline health-related information, including age, gender, occupation, educational qualification, and medical history of the past 6 months. The objective of this preliminary questionnaire was to evaluate their eligibility (and health condition) and interest in the study. Older adults (older than 60 y) who are generally healthy and able to walk >50% of the time around on their own or with the use of an assistive device (such as a walker) were included. In contrast, older adults with restricted mobility (like in a wheelchair >50% of the time) or conditions that may limit the use of devices, such as blindness and cognitive decline, were excluded. Aging people with severe cognitive dysfunctions were also excluded. The eligibility criteria included a set of subsectioned questionnaires to assess the participant’s health and mental health conditions, technological knowledge, and experience with modern technology to ensure their suitability for our study. From these observations, we found that most participants had no previous experience using technologically advanced devices like any smart wearable, and none had ever used a smartwatch.

Each participant was provided with an Oraimo Watch 4 Plus smartwatch after providing written informed consent to participate in the study. A detailed user guide was also supplied to ensure that participants clearly understood how to operate the device. At the time of the study, the device was commercially available at an approximate cost of US $20.06. The Oraimo Watch 4 Plus was selected due to its affordable price and the availability of standard health-monitoring features commonly found in other commercially available smartwatches.

#### Short-Term Survey

This survey aimed to determine short-term adherence and compliance to the protocol using the smartwatch device among the participants. Short-term surveys were conducted in 3 places after the participants were distributed the smartwatch for 14 days, with 27 participants from varying socioeconomic and educational backgrounds, as presented in Table 1. Among them, 15 out of 27 (56%) were male participants, and 12 out of 27 (44%) were female, with an average age of 60.55 (SD 7.38) years. In contrast, 13 out of 27 (48%) of participants belonged to a higher socioeconomic background, while 14 out of 27 (52%) belonged to a lower socioeconomic background. This survey involved a comprehensive questionnaire with 19 questions, including 15 multiple-choice and 4 short-answer questions. The following key questions were asked of the participants during the short-term survey:

1. How often do you wear your smartwatch?
2. How comfortable is your smartwatch to wear?
3. Have wearable health technologies helped you in achieving your health goals?
4. Did you find the screen size and text clear and easy to read?
5. Does using a smartwatch present any barriers concerning your religious, social, or cultural beliefs?
6. Did the device provide clear and simple instructions?
7. Did the device feel age-appropriate for you?
8. Have wearable health technologies helped you in achieving your health goals?
9. Which features do you like the best?
10. Which features do you feel uncomfortable with?
11. Which features are important to you in wearable health technology?
12. Would you recommend this device to other older adults?
13. Did you find the device frustrating to use?
14. What challenges do you face when using wearable health technology?
15. What improvements do you expect from this smartwatch?
16. How do you manage and interpret the data collected by your wearable health technology?
17. What kind of support do you expect from smartwatch manufacturers regarding religious use?

**Table 1.** Total number of participants, male and female ratio, mean age of participants, percentage of lower socioeconomic class participants, and percentage of higher socioeconomic class participants.

Survey type	Total participants, n	Male, n (%)	Female, n (%)	Age of participants (y), mean (SD)	Participants belonging to lower socioeconomic class, n (%)	Participants belonging to higher socioeconomic class, n (%)
Short-term survey	27	15 (56)	12 (44)	60.55 (7.38)	14 (52)	13 (49)
Long-term survey 1	19	15 (79)	4 (21)	65.00 (8.46)	7 (37)	12 (63)
Long-term survey 2	18	16 (89)	2 (11)	63.11 (5.26)	9 (50)	9 (50)
Long-term survey 3	18	15 (83)	3 (17)	63.16 (5.20)	10 (56)	8 (44)
Focus group interview 1	13	9 (69)	4 (31)	65.08 (9.59)	6 (46)	7 (54)
Focus group interview 2	12	10 (83)	2 (17)	64.75 (8.73)	7 (58)	5 (42)

#### Long-Term Survey

After completing the short-term survey, the long-term survey had to be proceeded with fewer participants. The participants of one of the old-age homes were excluded from the survey due to internal management issues at the organization, leading us to proceed with just one old-age home’s participants and a group of janitors. Also, a few participants from these 2 groups felt disinterested in continuing after the first DP of the long-term survey, primarily due to health issues and inability to adhere to the study guidelines. The first DP, long-term survey-1, was conducted with 19 participants, while long-term survey-2 and long-term survey-3 were conducted with 18 participants due to the sudden death of one of the participants from long-term survey-1. Each long-term survey was conducted at an interval of 10 days. Therefore, this survey section lasted for 30 days. As described in Table 1, the long-term survey-1 was conducted with 15 out of 19 (79%) male and 4 out of 19 (21%) female participants, 12 out of 19 (63%) belonging to a high socioeconomic background, and 7 out of 19 (37%) from a low socioeconomic background. In contrast, long-term survey-2 and long-term survey-3 were conducted with 16 out of 18 (89%) male participants and 2 out of 18 (11%) female participants. Additionally, a minimum of 8 out of 18 (45%) of the participants belonged to high socioeconomic backgrounds, while the other 10 out of 18 (56%) were from low socioeconomic backgrounds. The survey questions of the long-term survey were correlated to the short-term survey, and some variations in question types were used to improve assessment and analysis. The aim of this survey session was to find insights into usability factors of the smartwatch over a long-term period.

The key questions of the long-term survey included:

1. How often do you wear your smartwatch?
2. Which of the following health metrics do you track?
3. Which features do you like the best and are most comfortable with?
4. Which feature of the smartwatch do you find uncomfortable or dislike the most?
5. Rate how comfortable your smartwatch is to wear (out of 5).
6. Does using a smartwatch present any barriers to your religious, social, or cultural beliefs?
7. Did the device provide clear and simple instructions?
8. Has there been any technological challenge in using or understanding the watch?
9. Did the device feel age-appropriate for you?
10. Has the smartwatch helped you in achieving your health goals?
11. What challenges do you face when using the smartwatch?
12. What improvements do you expect from this smartwatch?
13. What health parameters did you collect from the smartwatch?

#### Focus Group Survey

Based on the socioeconomic background, 2 focus groups were formed among the participants of the long-term survey. However, some participants did not show interest in participating in these sessions due to various reasons. Group A included participants from higher socioeconomic backgrounds, while group B included participants from lower socioeconomic backgrounds. The first focus group survey was conducted 14 days after completion of the long-term survey (round 3), and the second focus group survey was conducted 14 days after the first focus group survey. These focus groups were repeated in 14-day intervals to investigate if the participants’ responses were consistent. More importantly, the focus groups were stratified across socioeconomic backgrounds to understand if and how different backgrounds might influence their experiences with the survey. The first focus group survey was conducted with 13 participants, while group A comprised 7 out of 13 (54%) participants, and group B comprised 6 out of 13 (46%).

In contrast, during the second focus group survey, there was 1 dropout due to illness, consisting of 12 participants, with 5 out of 12 (42%) representing group A and 7 out of 12 (58%) from group B. Each focus group session was conducted for approximately 1 and a half hours at the primary location of the participants to ensure convenience and comfort. Participants were asked structured questions during the sessions, and their responses were collected individually. A casual conversation was facilitated alongside the structured format to explore their thoughts and expectations regarding wearable technologies. The entire session was audio-recorded with participant consent to capture all responses accurately.

### Ethical Considerations

This user study or randomized controlled trial to study the adoption behavior of smartwatches among older adults was approved by North South University’s IRB and Ethics Review Committee (IRB #2023/OR-NSU/IRB/1231). All the surveys and focus groups described in the study were performed within the guidelines and duration of the approved IRB. A consent form and a baseline health questionnaire were evaluated from each participant before the surveys were conducted. Moreover, participants had the choice to drop out of the study at any time point, which happened over the course of the study. The participants were also provided with contact information of the researcher team for any queries. All data were collected, stored, and analyzed in compliance with HIPAA (Health Insurance Portability and Accountability Act) guidelines and data privacy laws. The participants were recruited by consent on a pro bono basis, although they were gifted the smartwatches at the end of the study.

## Results

### Short-Term Survey

#### Overview

The first short-term survey of the study was conducted to determine compliance levels among the participants from varying socioeconomic and educational backgrounds. The survey was designed with a variety of questions mentioned in the short-term survey section. A significant variation in responses was observed.

Responses to the first question, regarding the duration of smartwatch usage, are portrayed in Table 2. Notably, except for 1 individual, all participants used the smartwatch for at least 3 hours daily, with most participants using it for some time (defined as 3‐4 h), most of the day, and all day, highlighting the interest of the participants in exploring the smartwatch’s features and usability.

**Table 2.** Daily smartwatch use duration among participants (n=25).

Daily use duration	Participants, n (%)
All the time	7 (28)
Most of the day, except while sleeping	6 (24)
Occasionally	4 (16)
Sometimes	7 (28)
Rarely or never	1 (4)

The second question examined the health features participants tracked. Most of the participants used or tried using multiple features. As illustrated in Table 3, the most frequently tracked features were heart rate monitoring (“Heart Rate”), step counting, and oxygen saturation level monitoring (SpO_2_). Less frequently tracked features included sleep quality, calorie burn, respiratory rate monitoring, and stress level monitoring. Participants indicated that “Heart Rate,” “Step Count,” and “SpO_2_” features were easier to access and less complex to use than “Sleep Quality,” “Calorie Burn,” “Respiratory Rate Monitoring,” and “Stress Level Monitoring,” which were described as more challenging to understand and monitor effectively.

**Table 3.** Participants who reported using each smartwatch feature.

Used feature	Participants, n (%)
Steps	14 (56)
Heart rate	16 (64)
Sleep quality	3 (12)
Calorie, burn	5 (20)
Respiratory rate	3 (12)
Stress	5 (20)
SpO_2_^a^	12 (48)
None	4 (16)
Time	4 (16)

a SpO_2_: oxygen saturation level monitoring.

The third and fourth questions addressed privacy concerns and workplace compatibility. In response to these questions, all participants expressed positive views, indicating comfort in using the smartwatch at their workplaces without privacy concerns.

Questions 5 and 6 explored the features participants liked and disliked. Heart Rate emerged as the most liked feature due to its simplicity and ease of use.

Comfort, addressed in questions 7 and 8, was another key area of investigation. As illustrated in Table 4, most participants reported the smartwatch as “completely comfortable,” while a smaller group consisting of 3 (15%) users found it “mostly comfortable,” and another group of 4 (20%) users felt “somewhat comfortable,” suggesting that participants generally did not encounter significant physiological discomfort while wearing the device.

**Table 4.** Number of participants reporting comfort levels during smartwatch use.

Comfortability	Participants, n (%)
Very comfortable	1 (5)
Somewhat comfortable	4 (20)
Mostly comfortable	3 (15)
Completely comfortable	12 (60)

The ninth question examined whether the smartwatch posed religious, social, or cultural barriers for older adult individuals. As illustrated in Table 5, a majority (20 participants) responded “not at all,” while 2 participants reported “slight” barriers, and 2 remained neutral. Only 1 participant perceived the watch as a large hindrance while performing religious, social, and cultural activities.

**Table 5.** Participant responses regarding barriers.

Response	Participants, n (%)
Very	1 (4)
Slight	2 (8)
Not at all	20 (80)
Neutral	2 (8)

Participants were also asked whether they found the smartwatch age-appropriate and if they would recommend it to others in their age group. As illustrated in Table 6, approximately 52% (n=13) of the participants considered the smartwatch suitable for their age and indicated they would use it for a longer duration in the future. In comparison, 8 (32%) participants expressed neutrality. On the contrary, 2 participants found it very age-inappropriate and declined to use it in the future, citing concerns about advanced technology for older people. Furthermore, 1 female participant (>70 y old) from an old-age home commented:

*At this age, we cannot use advanced technology like a smartwatch. We are older people, and we believe our instincts suffice to predict physical disorders. I trust natural death will come when it is time. No technology is appropriate to predict diseases early, nor can it serve us with physical complexity.*

**Table 6.** Participant responses on whether the smartwatch was compatible with their age.

Response	Participants, n (%)
Yes	13 (52)
No	4 (16)
Neutral	8 (32)

Another question explored whether the smartwatch helped participants achieve health goals. A total of 13 (52%) participants believed it was helpful, while 3 (12%) felt it could not independently establish health goals. Moreover, 9 (36%) participants expressed neutrality. Table 7 presents an analysis of these responses.

**Table 7.** Responses from perceptions of whether smartwatch use helped achieve personal health goals.

Response	Participants, n (%)
Yes	13 (52)
No	3 (12)
Neutral	9 (36)

The final question focused on whether participants found the smartwatch frustrating to use. As illustrated in Table 8, 15 (60%) participants responded negatively, 4 (16%) reported frustrations, and 6 (24%) remained neutral. Frustration was reported mostly because of the age-inappropriate user interface.

**Table 8.** Responses to whether the device was frustrating to use.

Response	Participants, n (%)
Yes	4 (16)
No	15 (60)
Neutral	6 (24)

#### Participant Comments and Insights During Short-Term Survey

The survey also captured unique insights from participants, particularly those with lower levels of education. For example, a janitor noted:

*After using the smartwatch, I often felt hungry and could eat larger portions. This is due to the smartwatch.*

[Janitor, 63 y old, primary school graduate]

Similarly, another participant of an old-age home resident noted:

*I had arthritis, but after using the smartwatch, the pain miraculously healed. I think the device worked like medicine.*

[Old-age home resident, 103 y old, with no formal education]

Another female participant (62 y old) from an old-age home reported that she felt nausea, dizziness, and joint pain whenever she used the smartwatch.

The overall responses of this survey phase highlight that both groups of participants were curious about the smartwatch. However, they faced some challenges while using it. According to the responses, Heart Rate, Step Count, and SpO_2_ were the easiest features to monitor. Also, the watch was comfortable overall and created no barriers while doing regular activities. However, a few interesting comments were received from participants with lower education and socioeconomic backgrounds. Also, a large portion of the neutrality in the usability of the watch in achieving health goals indicates that a group of participants was confused about the appropriate technical knowledge of using the watch. Many participants shared their complexity in understanding the watch’s functionality during the interview. The research team answered that confusion with proper care and ensured clarification about the issues.

### Long-Term Study

#### Overview

The given survey revealed some interesting trends in smartwatch use and responses from participants across 3 phases of the long-term survey. Unlike the former short-term survey, this one included a series of questions categorized into daily usage, comfort level, feature usage patterns, and expected improvements that participants made.

#### Daily Usage

**Figure 2.**
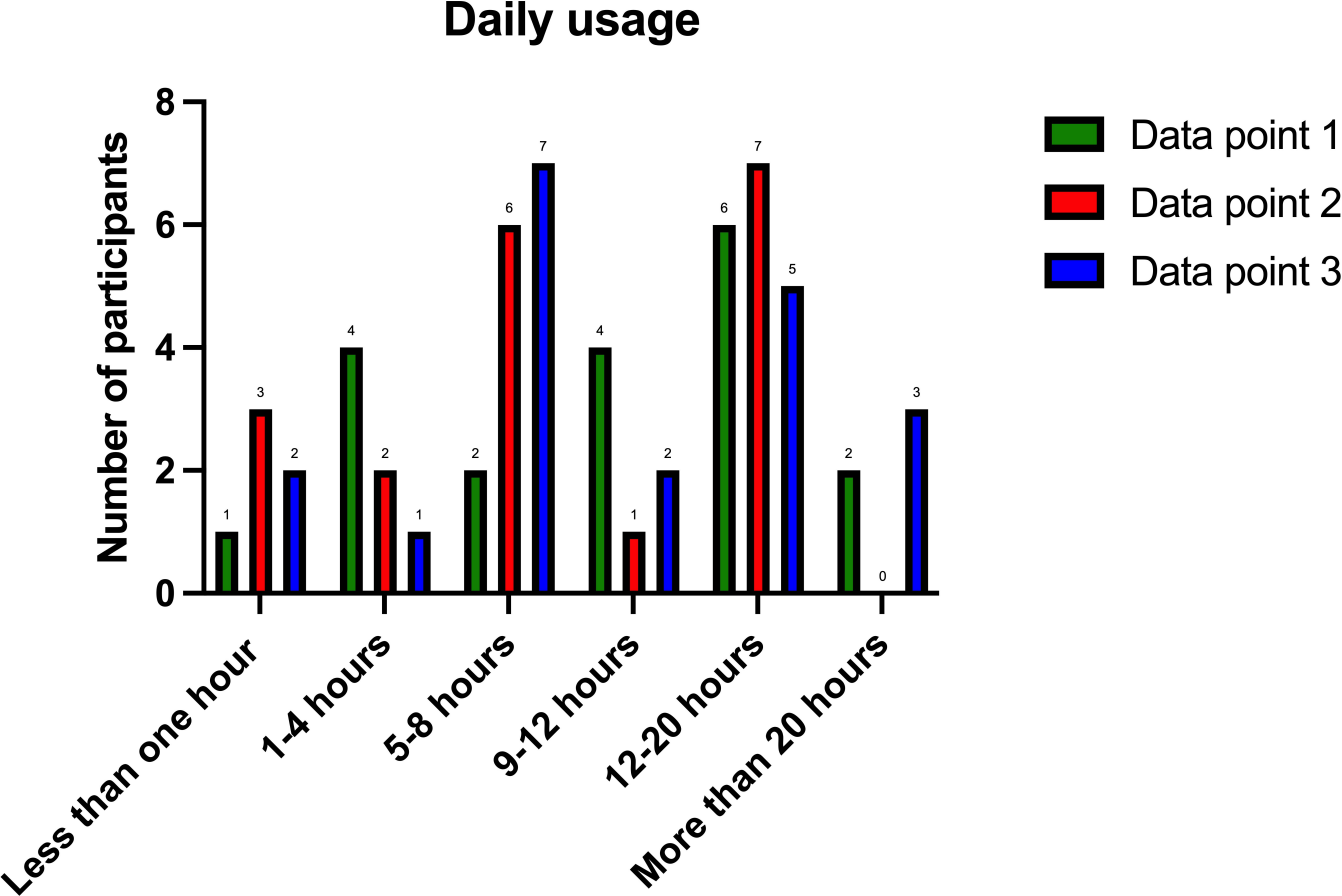
Grouped bar chart showing the number of participants reporting daily smartwatch use across 3 study time points (hours): <1 (n=1, 3, 2), 1‐4 (n=4, 2, 1), 5‐8 (n=2, 6, 7), 9‐12 (n=4, 1, 2), 12‐20 (n=6, 7, 5), and >20 (n=2, 0, 3).

As Figure 2 illustrates, 31.5% (6/19) of the participants used the smartwatch for 12‐20 hours daily during the first phase of the survey, thus being the most common usage duration; this was followed by 21% (4/19) of participants who used it from 9‐12 hours, and exactly the same percentage for those who said they used the smartwatch for 1‐4 hours daily. Smaller use of the smartwatch (daily periods exceeding 20 h) was reported by 10.5% (2/19), while a single participant reported not using it at all. Usage patterns continued shifting through the second phase, with 31.5% (6/19) of participants using the smartwatch for 5‐8 hours daily and 36.8% (7/19) for 12‐20 hours. Only 15.7% (3/19) of participants used the watch less than an hour daily, indicating that almost all participants engaged with the device to some degree. Finally, in the third phase, 20% (5/20) of respondents resumed using the smartwatch in the 12‐20 hours daily category, and 35% (7/20) used it between 5‐8 hours.

The results of the *t* test for daily usage, depicted in Table 2, showed a lack of significant variance in usage patterns across all phases; this is evidenced by *P* values ranging from .78 to ≥.99, which reveals that the overall time spent by the participants on the smartwatch remained relatively stable across the course of the study.

**Table 9.** Summary of paired *t* tests across 3 long-term study data points.

Survey dimension	Comparison	*t* test (*df*)	*P* value	Result
Daily usage	Data point 1 vs data point 2	0.00 (5)	>.99	Not significant
Daily usage	Data point 2 vs data point 3	−0.22 (5)	.83	Not significant
Daily usage	Data point 1 vs data point 3	−0.14 (5)	.89	Not significant
Rating on comfort	Data point 1 vs data point 2	0.30 (4)	.78	Not significant
Rating on comfort	Data point 2 vs data point 3	−0.17 (4)	.87	Not significant
Rating on comfort	Data point 1 vs data point 3	−0.07 (4)	.95	Not significant
Used features	Data point 1 vs data point 2	1.26 (8)	.24	Not significant
Used features	Data point 2 vs data point 3	−4.16 (8)	<.001	Significant
Used features	Data point 1 vs data point 3	−2.53 (8)	.03	Significant
Most liked features	Data point 1 vs data point 2	0.51 (8)	.62	Not significant
Most liked features	Data point 2 vs data point 3	−1.12 (8)	.30	Not significant
Most liked features	Data point 1 vs data point 3	−0.63 (8)	.55	Not significant
Clear instructions	Data point 1 vs data point 2	0.19 (2)	.87	Not significant
Clear instructions	Data point 2 vs data point 3	0.00 (2)	>.99	Not significant
Clear instructions	Data point 1 vs data point 3	0.09 (2)	.94	Not significant
Difficult to use feature	Data point 1 vs data point 2	1.96 (8)	.08	Not significant
Difficult to use feature	Data point 2 vs data point 3	−4.29 (8)	<.001	Significant
Difficult to use feature	Data point 1 vs data point 3	−2.31 (8)	.05	Marginally significant

#### Comfort Level Investigation

**Figure 3.**
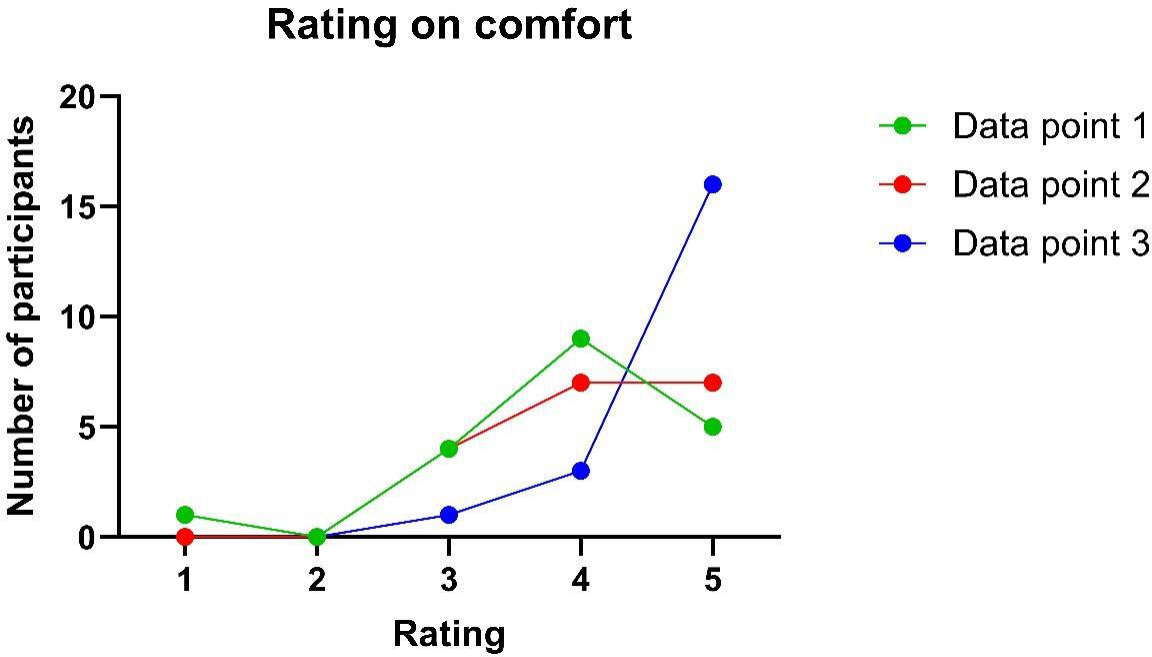
Line graph showing the distribution of comfort ratings (1=lowest to 5=highest) across 3 data points. The number of participants reporting higher comfort ratings (4-5) increased over time, with rating 5 rising at the final follow-up.

In fact, comfort levels improved a lot in the 3 phases, as portrayed in Figure 3. Initially, 47.3% (9/19) of the participants found the smartwatch comfortable, with only 26.3% (5/19) describing it as very comfortable. A small percentage, 5.3% (1/19), described it as highly uncomfortable since the features were complicated and the users did not have enough knowledge about them. However, in the second phase, no one reported discomfort, while 38.9% (7/18) found the smartwatch very comfortable, which marks the positive shift in user experience. In the third phase, 80% (16/20) rated the smartwatch as very comfortable, while only 1 participant reported it as fairly comfortable, amounting to 5% (1/20).

Furthermore, *t* test results of comfort ratings are insignificant among the phases, with *P* values ranging between .78 and .95 (Table 9). However, as revealed by the chi-square test, comfort perception improved dramatically over the survey period (Table 9), with a significant association—namely, *P*=.03 of phases with comfort ratings.

#### Investigation of Feature Usage Pattern

**Figure 4.**
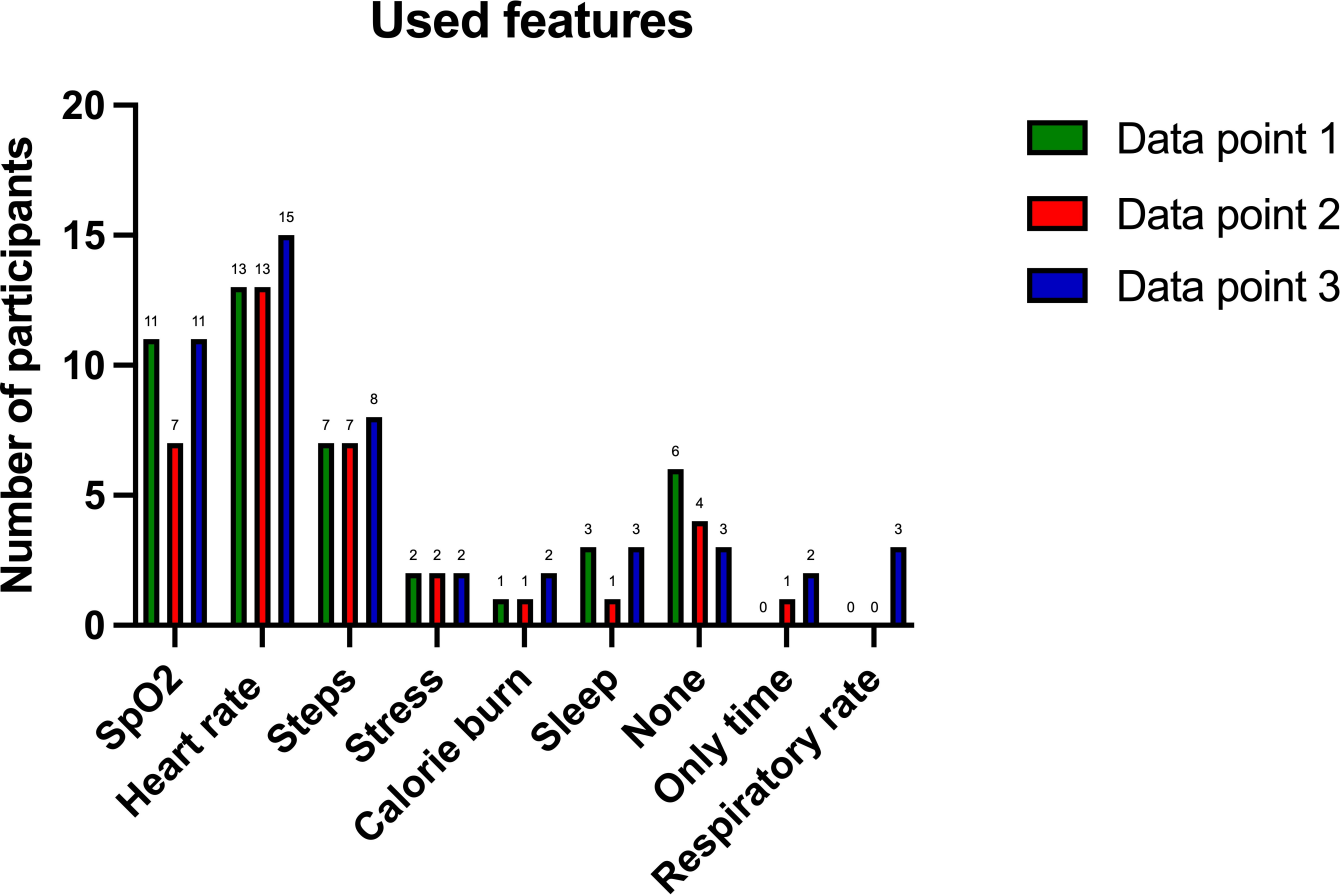
Grouped bar chart showing smartwatch feature use across 3 data points. Heart rate and step count were the most used features throughout, while other features were less commonly used and some participants reported using no features.

The usage of the features shows some fixed preferences along with some dynamic behavior, indicated in Figure 4 below. The most used feature in all phases remains heart rate monitoring, which shows an increased usage level from 68.4% (13/19) in phase 1 to 75% (15/20) in phase 3. The participants were primarily using other features such as SpO_2_ and Step Count, but with a small fluctuation in them. For example, usage of SpO_2_ reduced in phase 2 but increased in phase 3, which shows they were testing different features before making a preference.

The respiratory rate function, which had not been used in phases 1 and 2, gained acceptance among 15% (3/18) of participants in phase 3, which indicated increased interest in advanced functions with time as participants got accustomed to using the device.

The results of the *t* test indicated a significant change in used features over time, especially when comparing DP2 and DP3 (*P*<.001) and another significant change when comparing DP1 and DP3 (*P*=.03), which indicated that people have gotten used to using the smartwatch features.

**Figure 5.**
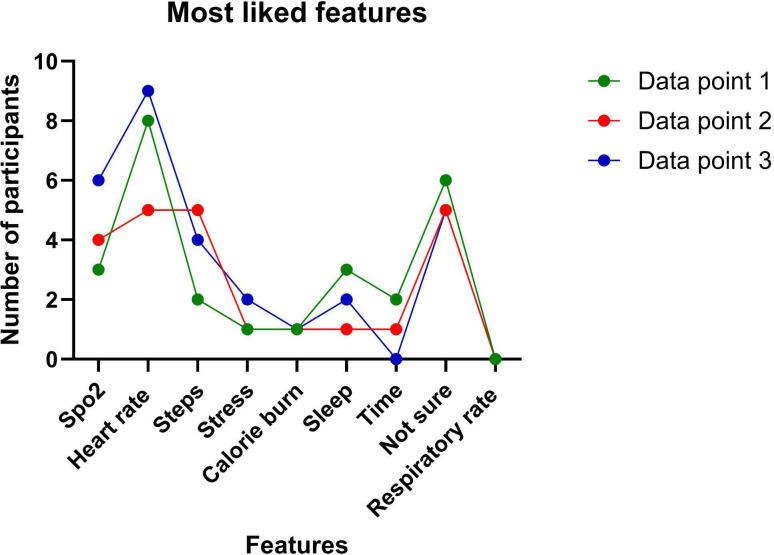
Line graph showing the number of participants selecting comfort ratings from 1 (lowest) to 5 (highest) at 3 measurement points. Comfort ratings shifted upward over time, with the highest rating (5) becoming most common at the final follow-up.

Besides usage, another area that we have explored is the most liked features in each stage of our survey, which can be seen in Figure 5. Heart Rate remains among the most liked features in all phases. As seen in Table 2, *t* test analysis for most liked features did not reach significance in the comparisons of DP1 with DP2 with a *P* value of .62, DP2 with DP3 with a *P* value of .30, and DP1 and DP3 with a *P* value of .55 for Heart Rate.

For example, in an attempt to capture features such as SpO_2_, the participants recorded a higher preference in the third phase relative to the first phase, as can be noted by the SD recorded in Table 9, which stood at 1.24722 (mean 4.33). Some of these features, such as SpO_2_, were thus better appreciated when participants were more conversant with the device. Other functions, such as tracking and respiratory rate measurement, were well-received but demonstrated inconsistency in preference, especially in the early phases.

What is interesting, however, is the fact that respiratory rate tracking was considered hard to use but gained acceptance over time, which is in line with the increased usage of advanced functionality in total. The nonuse of some functions in the first phase, with 31.6% (6/19) of participants not using any functions, highlights the learning curve involved in using smartwatches in general, which had declined substantially in phase 3. The above findings indicate how preferences in features change over time, with Heart Rate being a continually popular choice, given other features, such as SpO_2_ and respiratory rate monitoring, becoming more popular with increased familiarity.

#### Clear Instruction

Several trends were observed based on the feedback obtained from the participants with regard to the clarity of instructions, as presented in Table 10. At the beginning, 78.9% (15/19) found it simple and clear; hence, the reason for the easy adoption. This trend flipped as users continued using the application to explore features that became more complicated. In the second and third phases, the participants began to veer toward the neutral side concerning clarity. This is depicted by the high SD obtained in “Yes” (mean 12, SD 2.44) and “Neutral” (mean 4.33, SD 2.86), indicating that respondents do change with increased usage upon exploring more features. These results stress the need for increasing the clarity of instructions as users proceed to more complex features.

**Table 10.** Perceptions on clear and simple instructions across data points.

	Data point 1	Data point 2	Data point 3
Yes	15	12	9
No	3	2	1
Neutral	1	4	8

In Table 9, the chi-square test indicates a significant association (*P*=.04) between survey phase and participants’ opinion on instruction clarity, which signals the dynamic user perception as feature complexity increases.

#### Difficult to Use Features

**Figure 6.**
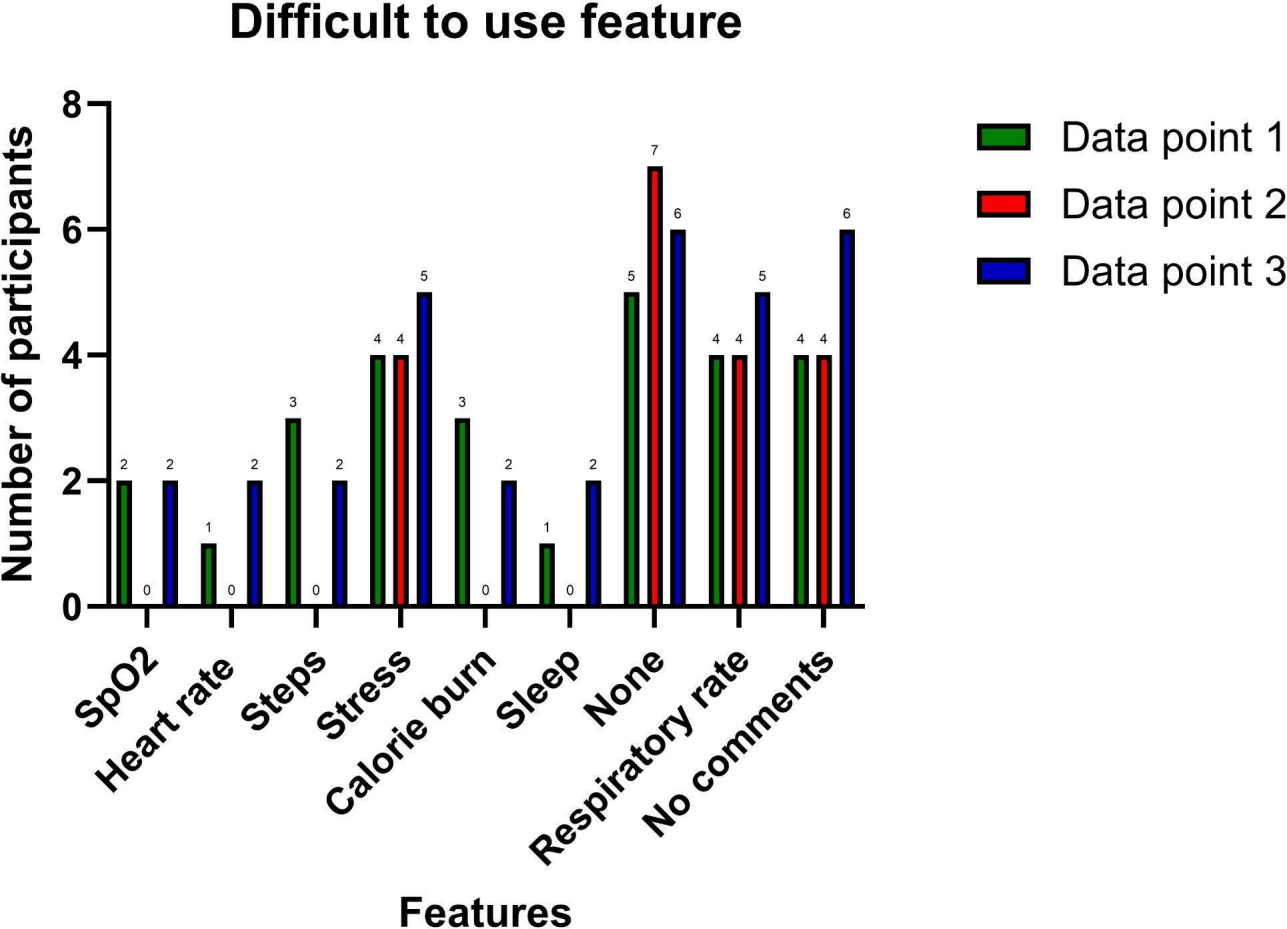
Grouped bar chart showing reported difficulty using smartwatch features across 3 data points. Most participants reported no difficulty, while some noted difficulty with specific features.

Figure 6 illustrates that the most difficult feature, especially in the third DP, is stress measurement. This would indicate a possible design or functionality issue with this feature. The same trend occurred for respiratory rate monitoring, which was considered a difficult feature through an increasing number of participants during the course of the experiment. Finally, on the opposite side of the spectrum, Heart Rate was the most favored feature and had no reports of difficulty throughout all points.

Furthermore, *t* test results indicate significant changes in the perceptions of difficulty regarding difficult to use features, where *P*<.001 for DP2 versus DP3 and *P*=.05 for DP1 versus DP3 support that design improvements are required in some features, such as stress measurement and respiratory rate.

#### Investigation of Expected Improvement

**Figure 7.**
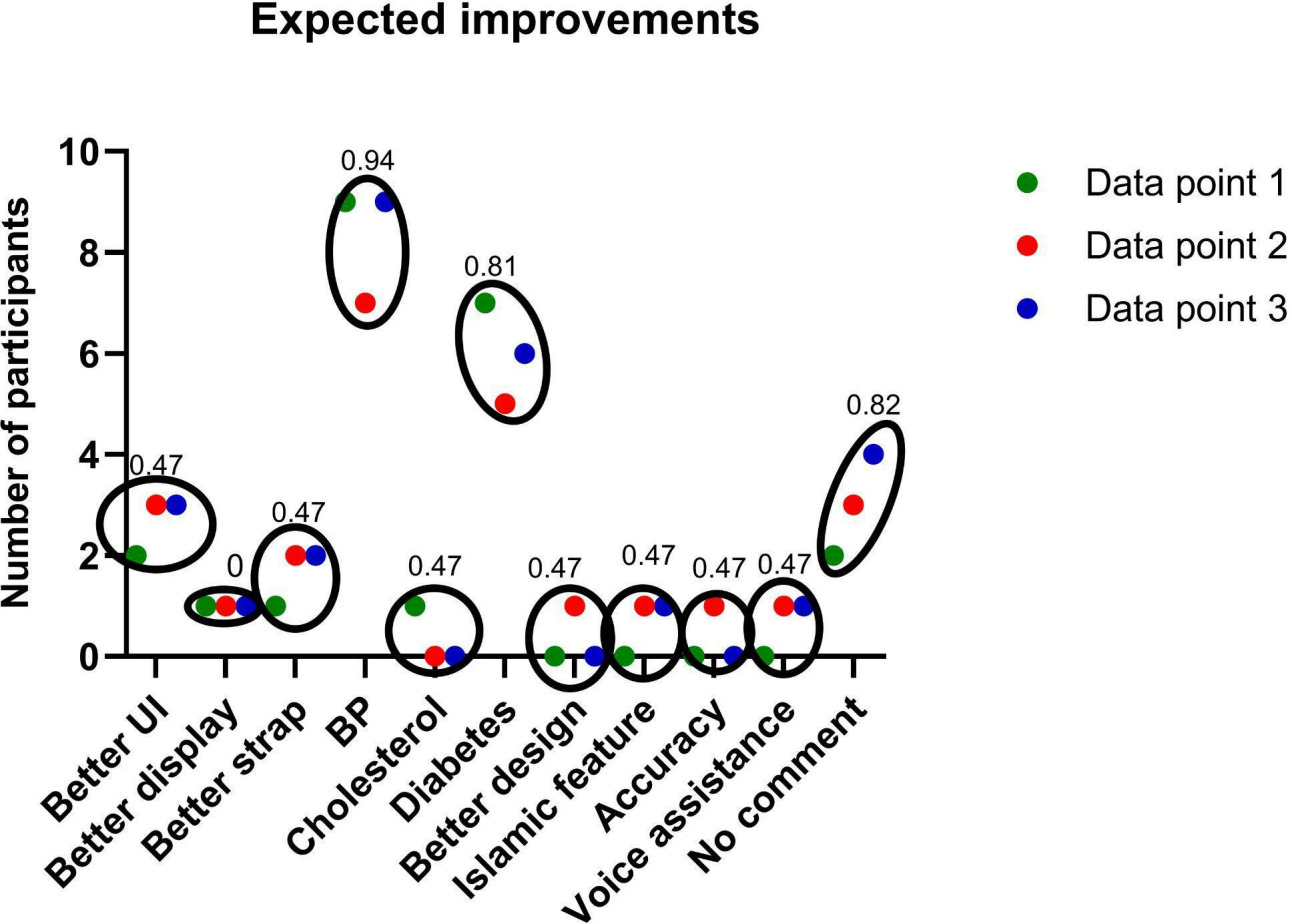
Scatter plot showing participant-suggested smartwatch improvements across 3 study time points (data point 1‐3). Points represent counts for each suggested feature, and labels indicate variability (SD) over time.

After long-term use of the smartwatch, adaptability and comfort were observed among the participants, as illustrated in Figure 7. Alongside, a trend was also observed in the expected improvements from the smartwatch (Figure 7). The data show that the most commonly suggested improvements were blood pressure and diabetes monitoring capabilities from the smartwatch, indicating that older people are mostly interested in critical health monitoring features. Interestingly, some participants initially had no comments on improvements but later suggested new features, though this shift was minimal. The overall low SD across all expected improvements confirms that user needs remained stable, reinforcing the necessity of designing a smartwatch specifically for older adults’ health monitoring.

### Focus Group Survey

#### Overview

The focus group interviews constituted the final phase of the study and were designed to provide qualitative insights that contextualize and extend the survey findings. Moreover, 2 rounds of focus group interviews were conducted using identical structured interview guides to enable both cross-group and longitudinal comparisons. Participants were stratified by socioeconomic status and educational background to explore how these factors influenced perceptions, adoption, and experiences of smartwatch-based health monitoring.

Group A comprised older adults residing in an assisted living facility, representing a higher socioeconomic and educational profile; all participants had completed at least a high school education, with the majority holding undergraduate or graduate degrees. Group B consisted of individuals from lower socioeconomic backgrounds with less formal education (below high school level), who were employed in custodial and maintenance roles. This stratification enabled a comparative examination of digital health adoption across differing social and educational contexts.

Qualitative responses were analyzed thematically, with dominant patterns reported alongside illustrative quotations. Descriptive response counts are included to enhance transparency, consistent with *Journal of Medical Internet Research *qualitative reporting guidelines.

#### Findings of Focus Group Interview 1

The focus group interviews took place in separate locations and under the same setting to gather similar perspectives. A total of 13 participants participated in the study, including 9 males and 4 females. Group A included 7 participants, of whom 4 were female and 3 were male. Group B consisted of 6 male participants. Both focus group interviews followed the same structured questionnaire to examine potential differences in responses and variability across the groups.

#### Perception of Smartwatches

Participants’ first impressions of smartwatches revealed clear group-level differences. Group A participants predominantly expressed positive attitudes, whereas group B participants more frequently articulated curiosity rather than immediate endorsement. A small subset of participants (2 from group A and 1 from group B) expressed skepticism or negative perceptions.

Qualitative analysis identified three emergent subthemes:

1. Perceived cognitive and physical burden,
2. Concerns about measurement accuracy, and
3. Skepticism toward replacing clinical expertise.

For instance, concerns about language and accessibility were raised by a group B participant (“I cannot read English texts well”), while doubts regarding clinical validity were voiced primarily by group A participants (“Technology cannot replace doctors”). Notably, these reservations were more pronounced among participants from higher socioeconomic backgrounds, despite their higher educational exposure to technology—implying deeper analysis and more cynicism in technology trust among users with higher education.

#### Usability of Smartwatches

**Figure 8.**
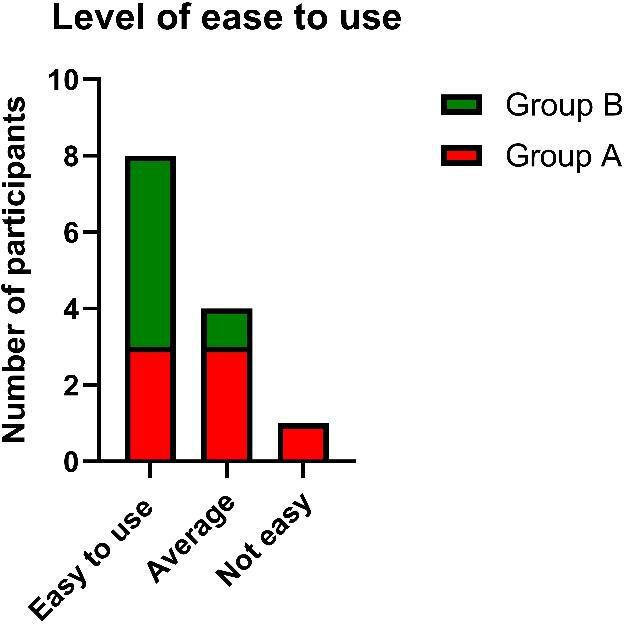
Stacked bar chart showing the number of participants rating the smartwatch as easy to use, average to use, or not easy to use, stratified by group (group A and group B).

When asked, “Did you find the smartwatch easy to use?” Most participants rated usability as “easy” or “average,” with only 1 participant reporting significant difficulty, as represented in Figure 8. Qualitative responses suggest that initial apprehension transitioned into gradual acclimatization, indicating a learning effect rather than persistent usability barriers. Participants emphasized novelty, visual familiarity with traditional watches, and perceived multifunctionality as positive features.

In light of this question, a further question was asked, “What did you love about it?” In response to this question, some participants answered,

*It is a new invention, and we were new users. At first, we were very excited after learning about such devices, and a positive impression of this device came to our heads. Furthermore, it looks quite similar to a normal watch, so we thought it offers more features than a normal watch; it is a good device.*

A participant from group B answered,

*I was curious when I found out about this. It looked smart, and I thought I would feel smart if I wore this watch. Also, since it offers many health-related topics, it would be a good tool for me.*

Therefore, this shows excitement in some older people about using new and “smart” technology, irrespective of their social belonging or understanding of the technology.

#### Comfort and Design Preferences

**Figure 9.**
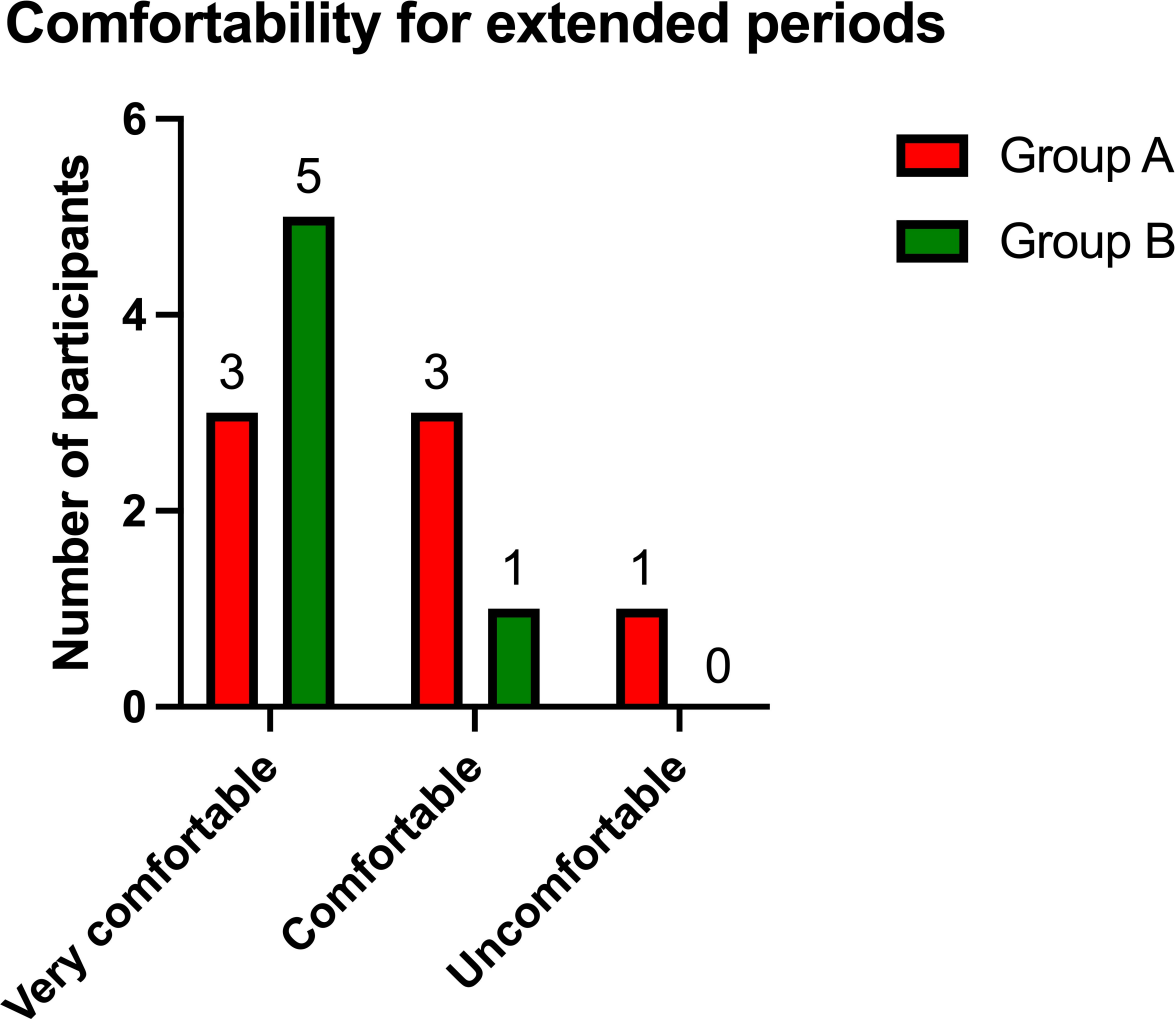
Stacked bar chart showing the number of participants who rated the smartwatch as very comfortable, comfortable, or uncomfortable for extended wear, stratified by group (group A and group B).

While overall comfort ratings were favorable, as illustrated in Figure 9, discomfort related to weight and strap design emerged as a recurring concern, particularly among group A participants. Participants expressed strong preferences for traditional materials (eg, leather or steel straps), suggesting that social acceptability and aesthetic continuity play a role in long-term adoption.

#### Data Sharing Preferences

**Figure 10.**
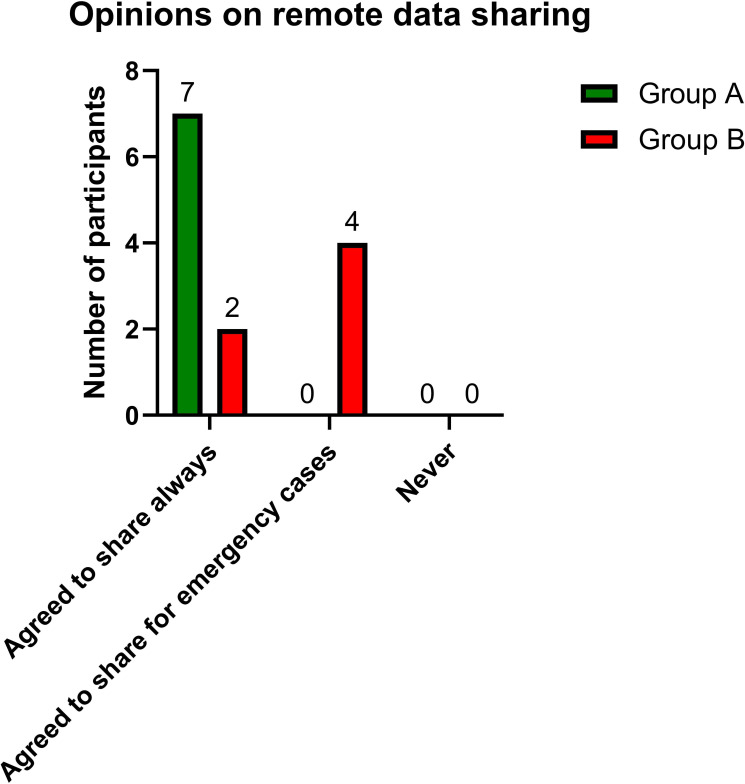
Bar chart showing the number of participants who agreed to share health data always, only in emergency cases, or never, stratified by group (group A and group B).

The question, “Would you like your data to be continuously and automatically shared with family members and/or designated caregivers?” received mostly positive feedback. As illustrated in Figure 10, all participants in group A responded positively. In contrast, 4 out of 16 participants from group B preferred that data sharing be enabled only during emergencies, while 2 participants expressed full support for continuous sharing. This divergence highlights differing expectations regarding privacy and data oversight between the 2 groups, as the less privileged (and less educated) group identified interpretability concerns and seemed more aware of not burdening their family with their health emergencies.

#### Perceived Accuracy of Health Metrics

In response to the question, “How accurate did the health metrics seem to you?” participants generally perceived health metrics as reliable. Limited engagement with data—not distrust—explained neutral responses, indicating that attention and interpretation, rather than sensor credibility, influenced perceived accuracy.

#### Focus Group Interview 2

The second focus group interview involved 12 participants (10 males and 2 females), with 5 participants in group A and 7 in group B. This interview was conducted 14 days after the first, enabling assessment of temporal stability and experiential adaptation.

#### Evaluation of Smartwatch Perception

Initial impressions in interview 2 closely mirrored those from interview 1, demonstrating thematic consistency over time. However, qualitative depth increased, with participants expressing more experience-based reflections rather than speculative judgments. Curiosity remained dominant in group B, while group A responses polarized slightly between positive endorsement and skepticism.

Compared with interview 1, negative perceptions were less frequent and more contextualized, suggesting that prolonged exposure mitigated abstract concerns while amplifying practical considerations.

#### Ease of Use: Reduced Variability Over Time

**Figure 11.**
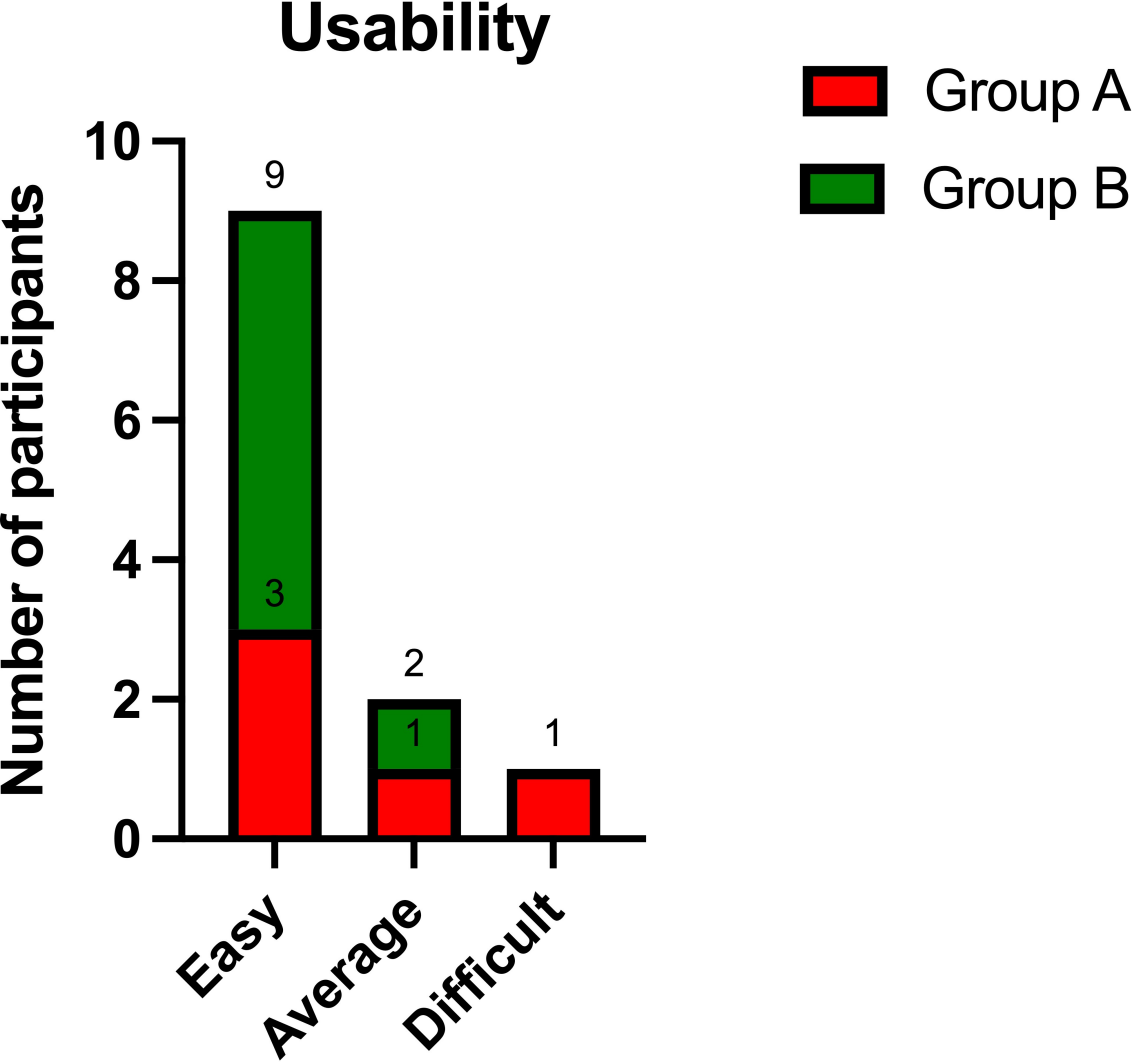
Stacked bar chart showing the number of participants who rated smartwatch use as easy, average, or difficult, stratified by group (group A and group B).

Usability ratings improved overall in interview 2, particularly within group B, as depicted in Figure 11. The reduction in “average” or negative usability responses indicates learning consolidation rather than mere tolerance. Difficulty reported by 1 older participant (88 y) underscores the influence of advanced age on interaction with wearable technologies, highlighting the need for age-adaptive interfaces.

#### Comfort: Consistency Across Interviews

**Figure 12.**
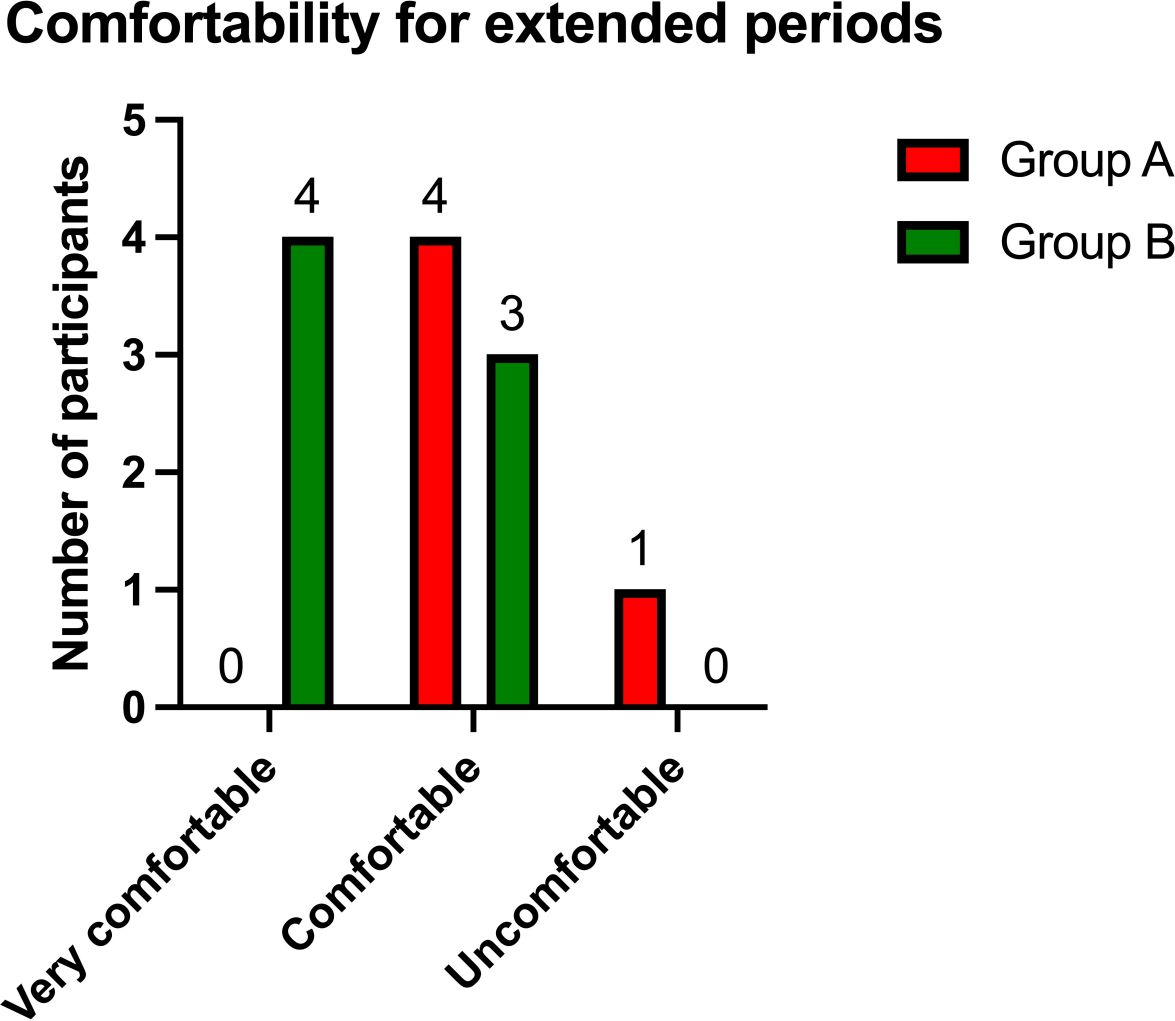
Stacked bar chart showing the number of participants who rated the smartwatch as very comfortable, comfortable, or uncomfortable for extended wear, stratified by group (group A and group B).

Figure 12 illustrates that comfort ratings in interview 2 remained stable relative to interview 1, reinforcing the robustness of this finding. Importantly, fewer participants raised new complaints, suggesting that comfort-related perceptions plateaued early and did not deteriorate with extended wear.

#### Data Sharing Preferences: Group Divergence Intensifies

**Figure 13.**
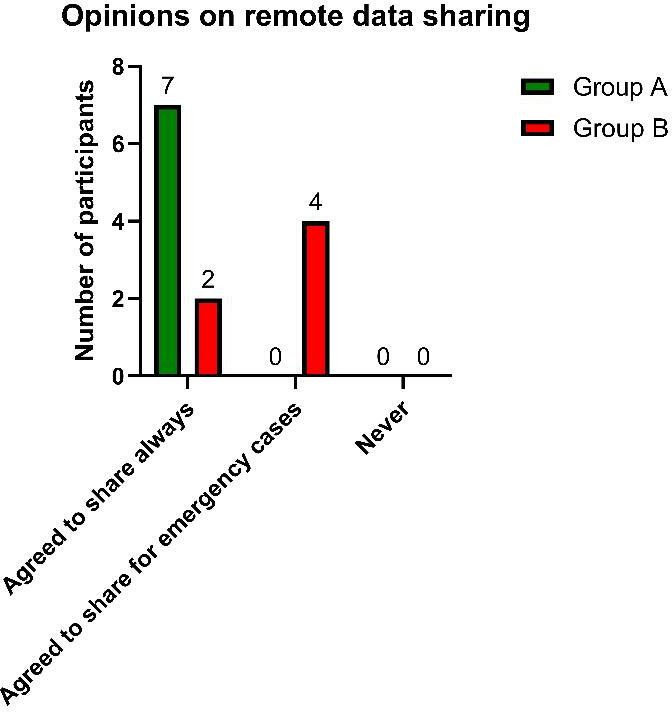
Bar chart showing the number of participants who agreed to share health data always, agreed to share only in emergency cases, or never agreed to share, stratified by group (group A and group B).

A clearer divergence in data sharing preferences emerged in interview 2, as illustrated in Figure 13. All group A participants supported continuous data sharing, whereas the majority of group B participants favored emergency-only sharing. Compared with interview 1, these preferences were articulated with greater certainty, indicating preference crystallization over time rather than ambivalence.

#### Perceived Accuracy of Health Metrics

Confidence in metric accuracy increased slightly in interview 2, particularly among group B participants. Overall, the responses were mostly neutral and a bit vague at this phase, again reflecting lack of attention rather than distrust, reinforcing findings from interview 1.

### Comparative Analysis and Analytical Power Considerations

#### Cross-Interview Thematic Stability

Across both focus group interviews, dominant themes, including usability adaptation, comfort acceptability, data-sharing conditionality, and perceived metric reliability, remained consistent. The repetition of themes across independent samples and time points supports thematic saturation, enhancing analytical power despite modest sample sizes.

#### Group-Level Comparative Strength

Although formal statistical power calculations are not applicable to qualitative focus group data, analytical power was strengthened through:

1. Replication of findings across 2 interview rounds,
2. Consistent group-level contrasts (higher vs lower socioeconomic status), and
3. Convergence between qualitative insights and descriptive survey trends.

The observed pattern, where participants from lower socioeconomic backgrounds adopted the smartwatch more readily and expressed fewer aesthetic or conceptual reservations, was robust across both interviews. This repeated contrast increases confidence that the finding reflects a genuine behavioral and perceptual trend rather than sampling variability.

### Limitations of Quantitative Inference

While descriptive counts are reported with consistency, the study is not powered for inferential statistical comparisons between groups due to the small number of participants. Group differences should therefore be interpreted as qualitative contrasts supported by frequency patterns, not as statistically generalizable effects.

### Implications for Mixed Methods Interpretation

The focus group findings provide explanatory depth for survey results, particularly by uncovering skepticism and aesthetic concerns that were not captured quantitatively. The consistency of these findings across 2 interviews enhances credibility and supports their use in informing design recommendations and future hypothesis-driven studies.

## Discussion

### Principal Findings

This study was guided by 3 overarching hypotheses. First (hypothesis 1), older adults’ smartwatch usage patterns would stabilize over time, with selective increases in feature engagement following continuous support, exposure, and training. Second (hypothesis 2), training and ongoing support would reduce perceived difficulty of use and improve long-term adoption. Third (hypothesis 3), socioeconomic and educational background would significantly shape usability experiences and data-sharing preferences.

Overall, the findings provide strong support for all 3 hypotheses.

First, consistent with hypothesis 1, basic health-monitoring features—particularly heart rate tracking—emerged as the most used and preferred functionality across all study phases, increasing from 68% (13/19) in the first phase to 75% (15/20) in the last phase of the final long-term survey. In contrast, more complex features, such as stress and respiratory rate monitoring, showed persistently lower engagement. This stable preference hierarchy underscores the importance of prioritizing core, easily interpretable health metrics in wearable interfaces for older adults, while introducing advanced features progressively as familiarity increases.

Second, longitudinal statistical analyses partially supported hypothesis 2. Across the 3 long-term study DPs, paired *t* test analyses (Table 2) indicated that most user experience dimensions remained stable over time. No significant differences were observed for daily usage, comfort ratings, most liked features, or clarity of instructions across any pairwise comparisons (all *P*>.05). In contrast, significant temporal changes were detected for feature usage, with increased differentiation between DP2 and DP3 (*t*_8_=–4.16, *P*<.001) and between DP1 and DP3 (*t*_8_=–2.53, *P*=.04). Perceived difficulty of use also showed change over time, reaching significance between DP2 and DP3 (*t*_8_=–4.29, *P*=.01) and marginal significance between DP1 and DP3 (*t*_8_=–2.31, *P*=.05). Complementary chi-square analyses (Table 9) largely supported these findings, revealing no significant associations between time point and most survey dimensions, except for clarity of instructions, which demonstrated a significant association with study phase (*χ*²_4_=10.3, *P*=.03), and a trend toward association for comfort ratings (*χ*²_8_=13.8, *P*=.09). Together, these results suggest overall stability in usage and satisfaction metrics over time, alongside meaningful evolution in feature engagement and perceived usability as participants gained longer-term experience with the system. Hence, our hypothesis was proved that training and support (as provided by the authors) helped the older participants to adopt and familiarize themselves with the smartwatch.

Third, findings strongly supported hypothesis 3, revealing pronounced socioeconomic differences in data-sharing preferences. Participants from higher socioeconomic and educational backgrounds expressed greater willingness to share health data continuously, whereas participants from lower socioeconomic groups predominantly preferred emergency-only sharing. Notably, reluctance to share data was often motivated not by privacy concerns alone, but by a desire to avoid burdening family members perceived as less technologically or medically informed. This highlights the need for flexible, customizable privacy and data-sharing controls that account for diverse social and familial contexts in LMIC settings.

Beyond these hypotheses, qualitative insights revealed a broader trajectory of adoption. Initial skepticism, particularly regarding accuracy and usefulness, gradually gave way to confidence and routine integration of the smartwatch into daily life. Participants consistently emphasized the importance of familiar physical design elements, such as leather or steel straps, suggesting that aesthetic familiarity plays a critical role in lowering psychological barriers to adoption among older adults.

### Key Contributions

This study makes several novel contributions to wearable health technology literature. First, it presents one of the few long-term, mixed methods usability evaluations of smartwatches among older adults in an LMIC context, specifically in Bangladesh. Second, the study uniquely incorporates 3 longitudinal survey cycles and 2 focus group interviews, enabling a nuanced understanding of adaptation over time rather than short-term acceptance alone. Third, by stratifying participants according to socioeconomic and educational background, the study reveals how adoption, usability challenges, and privacy preferences diverge across demographic groups—an aspect largely overlooked in previous work. Finally, the findings extend usability discourse beyond daily use to include cultural norms, family dynamics, and aesthetic preferences, offering a more holistic framework for designing inclusive wearable technologies for aging populations.

### Conclusion

This study investigated smartwatch usability, adoption, and perception among older adults through short-term surveys, long-term follow-up surveys, and focus group interviews. Findings demonstrate that while initial engagement was driven by curiosity and exploration, long-term use depended on comfort, usability, and relevance of features. Core health-monitoring functions, such as heart rate tracking, remained central to adoption, whereas complex features faced usability barriers despite training.

Over time, participants became more comfortable and confident with smartwatch use, validating the importance of continuous exposure, structured support, and intuitive design. Comfort-related concerns—particularly device weight and strap material, along with strong preferences for culturally familiar aesthetics, highlight the need for age- and context-sensitive hardware designs. Participants also expressed demand for features directly aligned with prevalent health concerns, including blood pressure monitoring, diabetes tracking, and caregiver-linked data sharing.

Socioeconomic and educational factors significantly shaped user experiences, particularly in relation to data-sharing preferences and perceived responsibility toward family members. These findings emphasize that successful wearable adoption among older adults, especially in the LMIC context, requires not only technical usability but also sensitivity to social values, privacy expectations, and cultural norms.

In conclusion, this study illustrates the evolving relationship between older adults and wearable health technologies, demonstrating both their promise and their limitations. By foregrounding longitudinal adaptation, socioeconomic diversity, and cultural context, the findings offer actionable design and deployment insights to support more equitable, acceptable, and sustainable adoption of smartwatches among aging populations.

### Limitations

A potential limitation of this study lies in the limited size and diversity of the participant sample. A significant proportion of the participants came from similar occupational backgrounds. Although the study targeted individuals from lower-income groups, it did not include any participants who were completely illiterate. Furthermore, due to unavoidable circumstances, such as illness or death, several participants either withdrew from the study or missed certain data collection points, resulting in some discrepancies in the dataset. To maintain data balance, some participants had to be excluded from the focus group sessions. Additionally, the sample exhibited minor gender biases. While the initial male-to-female ratio was satisfactory, a number of female participants later withdrew from the study due to health concerns. The absence of representation from more diverse gender identities and from individuals who are enthusiastic about technology but were not recruited may have impacted the inclusivity of the study. Future research should aim to include participants across a broader spectrum of demographics, regardless of gender, and with varying levels of technological literacy, including individuals who are entirely illiterate.

## Abbreviations

DP: data point
HIPAA: Health Insurance Portability and Accountability Act
IRB: institutional review board
LMIC: low- and middle-income country
SpO_2_: oxygen saturation level monitoring
